# Detection of single ion channel activity with carbon nanotubes

**DOI:** 10.1038/srep09208

**Published:** 2015-03-17

**Authors:** Weiwei Zhou, Yung Yu Wang, Tae-Sun Lim, Ted Pham, Dheeraj Jain, Peter J. Burke

**Affiliations:** 1 Integrated Nanosystems Research Facility, Department of Electrical Engineering and Computer Science, University of California Irvine, Irvine, CA, 92697 USA

## Abstract

Many processes in life are based on ion currents and membrane voltages controlled by a sophisticated and diverse family of membrane proteins (ion channels), which are comparable in size to the most advanced nanoelectronic components currently under development. Here we demonstrate an electrical assay of individual ion channel activity by measuring the dynamic opening and closing of the ion channel nanopores using single-walled carbon nanotubes (SWNTs). Two canonical dynamic ion channels (gramicidin A (gA) and alamethicin) and one static biological nanopore (α-hemolysin (α-HL)) were successfully incorporated into supported lipid bilayers (SLBs, an artificial cell membrane), which in turn were interfaced to the carbon nanotubes through a variety of polymer-cushion surface functionalization schemes. The ion channel current directly charges the quantum capacitance of a single nanotube in a network of purified semiconducting nanotubes. This work forms the foundation for a scalable, massively parallel architecture of 1d nanoelectronic devices interrogating electrophysiology at the single ion channel level.

Ion channel membrane proteins play a critical role in life and death^1^, and are known to be the target of 25% of available drug targets^2^. New methods of probing ion channel activity, especially those that can be scaled up to massively parallel high throughout sensing, as well as new electrode technologies that can be used for in-vivo sensing with high spatial resolution (down to the single ion channel level) can enable the study of electrophysiology in a broad range of applications. One such platform is an artificial cell membrane on solid substrate surface (supported lipid bilayer (SLBs))^3^,^4^,^5^. Ensemble measurements of ion channel currents are possible in this architecture on a variety of solid substrates, including Ag^6^, silicon^7^,^8^, SiO_2_^9^, indium tin oxide (ITO)^10^, and have been demonstrated as systems for the interrogation of single ion channels^11^. However, the spatial resolution of these systems is modest (100 × 100 μm electrodes have been demonstrated), and challenges for massively parallel scaling have not been addressed.

The potential of nanoelectronics technology to interface with electrochemistry and electrophysiology represents a clear opportunity for a scalable, massively parallel architecture of nanoelectronic devices interrogating electrophysiology at the single ion channel level. Although several methods of lipid bilayer attachment to nanowires^12^,^13^,^14^,^15^ and nanotubes^16^,^17^,^18^,^19^,^20^ have been demonstrated, similar to the bulk electrode interrogation, all the electrical measurements to date have been ensemble measurements of macroscopic numbers of ion channels resulting in an integrated electrical signal representing the net effect of all the ion channels in parallel^14^,^15^,^17^,^18^,^19^. Of this broad class of nanoelectronics systems, single walled carbon nanotubes (SWNTs)^21^ represent arguably the smallest and most appropriate model system on which to develop of models of ion channel –nanoelectronic interaction motifs and mechanisms that would be more broadly applicable to a wide class of nanoelectronic devices based on reduced dimensions, such as semiconducting nanowires^23^, 2d nanomaterials^24^,^25^, and any other nanoelectronic device with dimensions comparable to a single biological ion channel.

Here we demonstrate a simple robust platform using to measure individual ion channel currents through individual ion channel proteins in lipid bilayer membrane. (A portion of this work has already been presented in abstract form^26^,^27^,^28^). A simple physical model of the ion channel current directly charging the quantum capacitance of a single nanotube in a network of purified semiconducting nanotubes is presented, which provides an explanation to the observed current waveforms. In order to provide a biocompatible, hydrophilic surface with sufficient space for the insertion of ion channel proteins into the membrane without perturbation or denaturation from the nanotube or solid substrate^4^, we develop a polymer-cushion strategy by using three different surface functionalization schemes: phospholipid-polyethylene glycol (PEG), PEG-silane, and poly-L-lysine (PLL). All three schemes are shown to successfully generate a biocompatible hydrophilic interface between the lipid bilayer and the nanotube network. Using this system, we incorporate ion channel membrane proteins gramicidin A (gA), alamethicin, and a static biological nanopore α-HL (α-HL) into the lipid bilayer membrane. The nanotube electrodes are able to sense the dynamics of single ion channel pore opening (by sensing the ion channel current), as well as polymer blocking of the nanopore currents (in the case of α-HL, which is always open). While this demonstration is for carbon nanotubes, we believe this motif is more broadly applicable to a broad class of 1d nanoelectronic devices of semiconducting materials, e.g. silicon nanowires^23^, indium based nanowires^29^, or even optically active nanowires^30^. Together with our recent analogous work on 2d nanomaterial interrogation of single ion channels^25^, this work (using a 1d nanomaterial) forms the foundation for a scalable, massively parallel architecture of nanoelectronic devices interrogating electrophysiology at the single ion channel level.

## Results

### Preparation and characterization of semiconducting SWNT transistors

**Figure 1:**
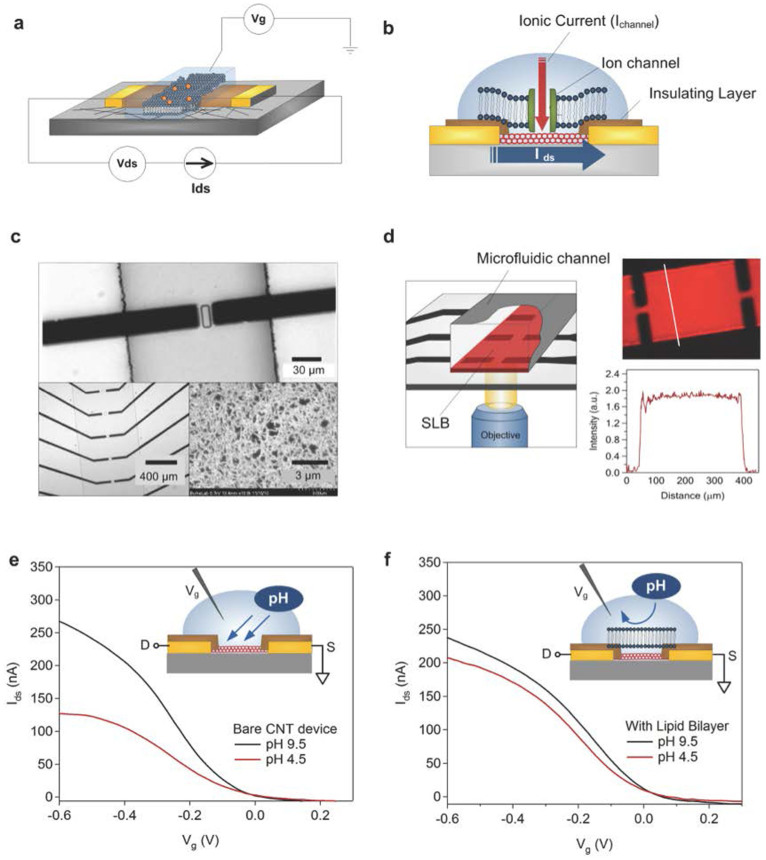
Schematic view of fabricated devices and pH response of devices. (a) Experimental set-up showing a nanotube device. (b) Schematic diagram of the device currents: Ion channel current (I_channel_) and source-drain current (I_ds_). (c) Optical images of a micrograph of source and drain contacts showing a window (top) and an array of SWNT transistor with source and drain pairs (bottom left) and an SEM image of nanotube networks (bottom right). (d) Left: Schematic of device set-up for SLB deposition (red); Right: fluorescence image of lipid bilayer; Bottom right: fluorescence intensity profile of the solid white line. (e) Transfer characteristics of a bare SWNT transistor without lipid bilayer coating with pH response and (f) Transfer characteristics of an SLB-coated-SWNT transistor with pH response.

The random network of purified, all semiconducting nanotubes used as the basis for our device is depicted schematically in Fig. 1(a) (see methods for detailed fabrication process). Briefly, deposition of the nanotube network is according to our recently published procedure^31^. Compared with 2d graphene, our 1d SWNT network provides sub-monolayer surface coverage. Therefore, it becomes essential to obtain a uniform SWNT film with a proper density on the wafer surface for using SWNTs as electrodes to detect individual ion channel activity. In this work, we kept the density of nanotube networks 5–10 nanotubes μm^−2^ to preclude the alteration of device performance by variation in the density of nanotubes (Fig. 1c (bottom right) and Fig. S4), which can be easily controlled by tuning the concentration of nanotube ink solution^31^. As shown in Fig. S4, the SWNT network is uniform in the device channel area.

**Figure 2:**
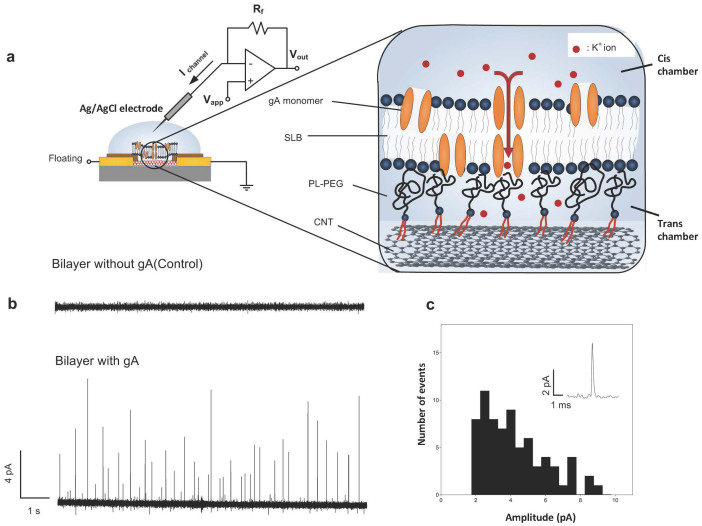
Ion channel recordings. (a) Schematic of ion channel recording set-up showing a simplified equivalent circuit of patch clamp amplifier. Inset: The interface between SLBs with gA and CNTs linked with PL-PEG polymer linkers. (b) Ion channel current trace (top) before gA insertion (bottom) after introduction of gA (cutoff frequency: 1 KHz, sampling: 20 KHz, V_app_ = +50 mV). 100 mM KCl in 1× PBS solution was used for recording. (c) Histogram of ion channel events; Inset: a zoomed-in typical event.

**Figure 3:**
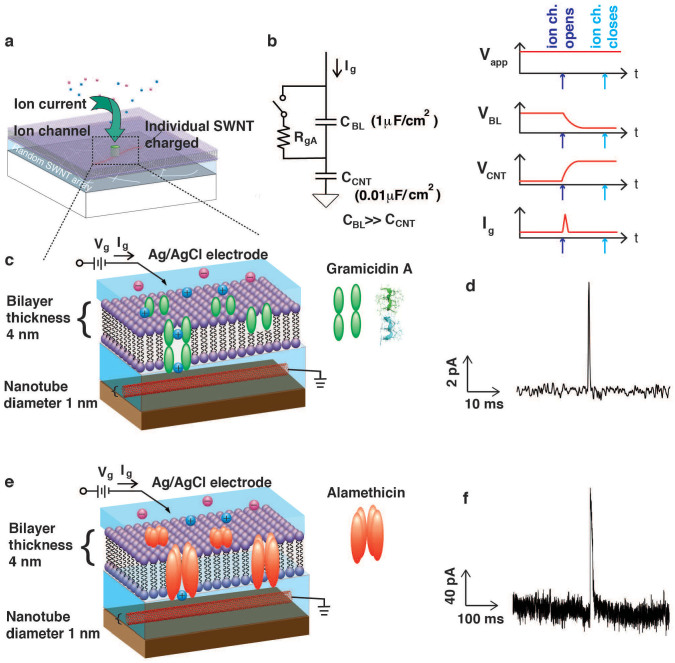
Circuit and schematic interpretation. (a) Schematic 3d representation showing a single ion channel opening, and the current charging a single nanotube in the matt. (b) Circuit and time domain behavior of essential elements of ion channel opening and closing. (c) Schematic representation of gramicidin opening and closing. The nanotube and lipid bilayer are to scale. The functionalization layer is not shown, and represented in the next figure. gA protein structure from PDB ID: 1 grm^51^. (d) Representative current spike with gA. (e,f) As for (c,d) but with alamethicin. 100 mM KCl in 1× PBS buffer was used for recording.

How is the density of SWNTs related to the ion channel density? For the same (molar) concentration ratio of gA to lipid ratio used in this work, previous research has found a channel density of order 10^9^ channels/cm^2^, or a mean channel to channel spacing of 300 nm^32^. In comparison, our SWNT density is 5–10 nanotubes μm^−2^, with each nanotube of order 1 μm in length. In addition, the ion channel geometry is comparable in size to the diameter (but not length) of a SWNT (of order nm) (Fig. 2a, 3c and 3e). Thus, it is expected that only one nanotube is loaded upon opening of an ion channel.

Our previous study^31^ showed that the density of nanotubes is highly related to device electrical properties such as mobility and on/off ratio. The nanotube network is contacted with metal electrodes (source/drain), and, for the delivery of electrolytes, integrated into a polydimethylsiloxane (PDMS) microfluidic channel. Figure 1c (top) shows an optical image of a representative nanotube transistor with a 20-μm gap metal contact and a microfluidic channel on the top. Figure 1c (bottom left) is a nanotube device array picture. In order to suppress unwanted electrochemical currents from the metal contact electrodes into the electrolyte solution, an insulating passivation layer is deposited which fully covers the metal electrodes while leaving most of the nanotubes exposed to the solution. In figure 1c (top), a 30 × 15 μm^2^ photoresist window was opened in the middle of the source/drain gap by photolithography. In our experiments, the exposed nanotubes are either directly in contact with the solution, or covered with supported phospholipid, 1, 2-Dioleoyl-sn-glycero-3-phosphocholine (DOPC) or 1,2-diphytanoyl-*sn*-glycero-3-phosphocholine (DPhPC) bilayer, either as is, or containing additional embedded transmembrane proteins. In all cases, the electrolyte can be used as a gate to control source-drain current; the gate voltage is applied to the electrolyte *via* an Ag/AgCl reference electrode (Fig. 1(a)). A side view (Fig. 1(b)) of the device schematic shows the two current paths that we are interested in investigating: the ion channel current (I_channel_) through an ion channel and source-drain current (I_ds_) through the nanotube transistor.

### Forming lipid bilayer on SWNT transistor surface with polymer functionalization

**Figure 4:**
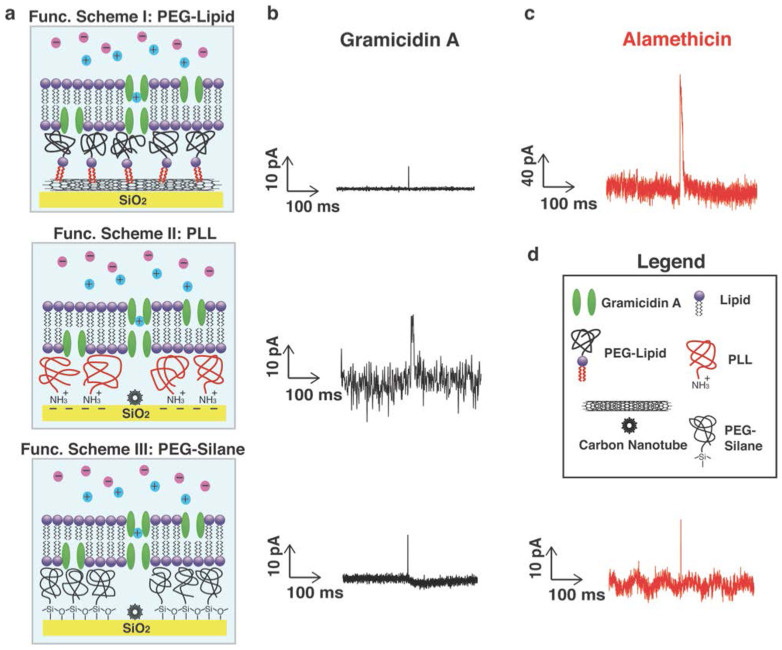
Functionalization schemes. (a): PEG-lipid, PLL, and mPEG-silane functionalization schemes. (b) Current traces for gA using all 3 schemes. (c) Current traces for alamethicin using PEG-lipid and mPEG-silane functionalization schemes. (d) Legend. 100 mM KCl in 1× PBS buffer was used for recording in the experiments using PEG-lipid and mPEG-silane functionalization schemes. 1 M CsCl in 1× PBS buffer was used for recording in the experiment using PLL functionalization scheme.

The schematic in Fig. 1 (d, left) shows the experimental set-up used for observing lipid bilayers that is confined by a microfluidic channel. In this paper, we use the vesicle fusion method to deposit SLBs on a quartz wafer surface. A hydrophilic surface is a prerequisite for forming a lipid bilayer. Lipid vesicles can effectively rupture and transform to a planar membrane on a fresh piranha treated quartz surface after incubation (Fig. S5a), whereas a continuous lipid bilayer cannot spontaneously form on high-density SWNT network due to its hydrophobic surface (Fig. S5e). The lipid membrane coverage is even lower when incorporated with ion-channel proteins (Fig. S5f). In order to achieve continuous SLBs on the nanotube network by the vesicle fusion method, we examined SLB deposition with various substrate conditions functionalized with different moieties. We developed three functionalization schemes, all of which were successful in providing a hydrated interface between the lipid bilayer and the nanotube network. The first functionalization scheme uses the hydrophobic tail of a lipid attached to a PEG polymer (MW 2000), and was motivated by the work of Dai, et al^33^, who found that PEG lipid (DSPE-PEG 2000)^33^ was effective in solubilizing nanotubes. The hydrophobic lipid tail can non-covalently bind to the hydrophobic sidewalls of the carbon nanotubes, providing a strong anchor for the PEG polymer cushion to which the lipid bilayers would attach after vesicle fusion (Fig. 4a (top)). (In principle, as an alternative, the hydrophobic moieties of some peptides^34^, DNA, and even lipids in organic solvents^22^ can also be used to bind to the hydrophobic sidewalls of SWNTs.) A second method was motivated by studies of Huang^19^, who showed that PLL could form an effective polymer cushion between carboxylated nanotubes (with hydrophilic dangling bonds and -COOH groups) and lipid bilayers. In our experiments, this method was equally as effective. We speculate that this was probably because positively charged PLL also can be electrostatically absorbed onto the negatively charged SiO_2_ substrate (Fig. 4a (middle)). While both of these non-covalent methods were successful, we further sought a more rugged attachment of the PEG polymer cushion to the substrate using covalent silanol coupling chemistry. Noncovalent attachment of lipid bilayers to surfaces can be fragile with lifetimes of order hours, whereas covalently attached lipid bilayers can last for months or even a year^35^. To meet this challenge, we developed a process based on silanol chemistry to covalently attach a commercially available PEG (MW1000)-silane to the SiO_2_ substrate (Fig. 4a (bottom)). PEG-lipid modifies carbon nanotube surface through the hydrophobic interaction between lipid tail and carbon nanotube wall and PLL modifies quartz wafer and carbon nanotube surface through being electrostatically absorbed onto negatively charged SiO_2_ surface and carboxylated carbon nanotube surface, while PEG-silane is covalently bonded on quartz wafer surface through silane anchor. Therefore, PEG-silane cushion is the most robust system in the three functionalization schemes. All three of these methods have been demonstrated in this paper to form an effect polymer cushion for the attachment of lipid bilayers in the vesicle fusion method (shown in Fig. S5b, c and d).

Fig. 1 (d, top-right) shows a representative fluorescence image of SLB films (red) incorporated with gA displaying two arrays of source and drain contacts as black rectangles after PEG-lipid functionalization. The uniform fluorescence intensity profile (bottom-right) for the region of solid white line in the fluorescence image indicates that the SLB films are smooth and continuous, and the surface modification was successful without defects.

In order to evaluate the effect of the bare SLB (prior to incorporation of membrane proteins) on the nanotube electrical properties, we compared the depletion curve of the nanotube device before and after deposition of the SLBs that contain no ion channels. The device with CNTs without coated SLB showed a sensitive response to changes in pH level of solution^36^,^37^ while obvious decreases in sensitivity were observed from the device shielded with SLBs (Fig. 1(e, f)). This clearly demonstrates that the SLB is forming an effective electrical barrier between the bulk electrolyte and the nanotube device.

### Detection of single ion channel activity by SWNT network electrodes

After establishing a method for lipid bilayer functionalization, we aimed to examine the current directly through ion channels using the nanotubes as electrodes. To reconstitute gA in SLBs, gA in ethanolic stock was premixed with DOPC chloroform solution before solvent evaporation process, while alamethicin and α-HL both inserted to preformed SLB by directly adding to *cis* chamber (top) from their ethanolic stock (see Methods). For the ion channel recording from our devices, we used a patch clamp amplifier that has an operational amplifier with negative feedback that automatically maintains a constant applied potential by injecting a displacement current into the electrode^38^ (Fig. 2(a)). A two-electrode system was used for ion channel activity recording. A silver/silver chloride (Ag/AgCl) wire electrode was inserted into buffer solution over the *cis* chamber of the device that is made by PDMS bonded on the chip surface. The voltage was applied to *cis* chamber through Ag/AgCl electrode connected to the headstage of patch clamp system. The reference electrode is the SWNT network on the trans-side, which was contacted with a tungsten probe through a source or drain electrode pad. All the current measurements using patch clamp system were carried out inside a Faraday cage to shield the device from external electric fields. Potassium chloride or cesium chloride solutions were used for measurement electrolytes (see supplementary information for detailed concentration for each experiment). Unlike a conventional Ag/AgCl reference electrode, the SWNT network electrode generates only minute Faradaic currents within the small voltage window we used. Many previous studies on electrolyte-gated semiconducting SWNT transistors^39^,^40^,^41^ and electrochemistry of SWNTs^42^,^43^,^44^ have demonstrated very low electron transport (Faradaic current) from SWNTs to electrolyte solution unless there are redox pairs in the electrolyte solution^42^. When the applied gating voltage is within ±1 V, the quantum capacitance of carbon nanotubes dominates in the whole interfacial capacitance because it is an order of magnitude less than electrostatic double layer capacitance that is in series with^43^,^44^. Therefore, the electrolyte solution, as a high efficient gate, is capacitive gating SWNTs and not allowing a significant dc current from the nanotube network to the electrolyte solution. If the Faradaic current is small, how, exactly, does current flow from the SWNT, through the ion channel, and into the Ag/AgCl electrode? This question will be answered in the interpretation/discussion section.

The interface between the nanotubes and the SLB is graphically depicted in Fig. 2(a) showing that the SLBs are linked to CNTs via PL-PEG polymer linkers which also acts as a spacer enabling enough room for membrane proteins preventing denaturing from the direct contact to the bottom surface^6^. Fig. 2 (b, c) shows ion channel current waveforms and histograms measured. It is important to note that this is the current through the lipid bilayer, using the nanotube network as the reference electrode to source/sink the current. The inset shows the control current trace from the device coated with only SLBs before the addition of gA. No current spikes were observed from this experiment demonstrating that no ion transport through the lipid bilayer occurs, as well as showing that the device is completely shielded by the lipid bilayer. As the spikes occur only after introduction of gA, this is clear demonstration of current through a nanopore in our SLB device. In addition, while it is possible that a given current spike corresponds to many gA pores, we find the most plausible explanation that the current spikes are occurring due to the opening and closing of single ion channels. At a given applied voltage (+50 mV), the measured current is ~ a few pA, which is similar to the value of the single channel opening/closing reported by literatures^11^,^45^. This is the first measurement of single gA channel activity in SLBs ever demonstrated on SLBs on 1d nanowire/nanotube-based electrodes.

**Figure 5:**
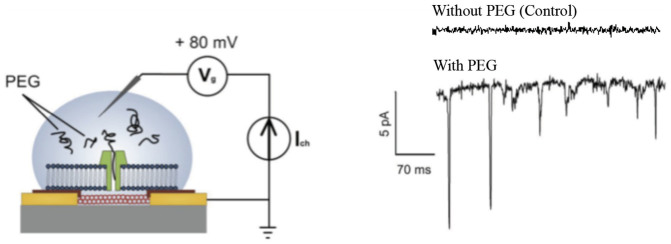
Ion channel recordings. Polymer (PEG) translocation through α-HL showing PEG polymer blocking of the nanopore currents. Inset: schematics of device side-view during the measurement. 100 mM KCl in 1× PBS buffer was used for recording.

We then introduced another voltage-gated ion channel (alamethicin) into our lipid bilayer platform, which in certain models has uncertain number monomers to form channels during the measurement, typically resulting in multiple conductance levels^45^. In Fig. 3, we show the current spikes from gA and alamethicin laden bilayers. The current spikes from the alamethicin incorporated bilayers we observed are of order 100 pA, which is in good agreement with the known properties of alamethicin in suspended lipid bilayers^45^. This indicates that the current spikes are indeed measuring individual ion channel currents, whose amplitude is consistent with the known conductance of the same ion channels in suspended membranes. The observed phenomena are robust and independent of the functionalization scheme used (Fig. 4 a,b,c and Fig. 5), as long as a polymer cushion is in place between the nanotube network and the lipid bilayer.

While the individual spikes are clearly related to the introduction of the gA, the waveform is different than that observed when single gA channels are measured with black lipid membranes (BLMs). In BLM, the opening and closing to the gA pores has a time constant of about 1 second, due to the 6 hydrogen bonds between the dimers causing long-lived dimer states^45^. In these classical electrophysiology experiments, the current *vs* time waveform occurs in steps, rather than spikes^45^. Each step corresponds to the opening or closing of a single gA pore, but since all realizations of the pore have the same geometry and conductance, the current steps are uniform from step to step. In contrast, a histogram of the heights of the current spikes we observe (Fig. 2c) shows no apparent typical current, indicating heterogeneity among the pores or their electronic coupling to the individual nanotube, which may vary with the distance from pore to nanotube. Thus, the geometry of our experiments clearly causes different amplitudes due to the current of gA in SLBs than in suspended membranes.

**Figure 6:**
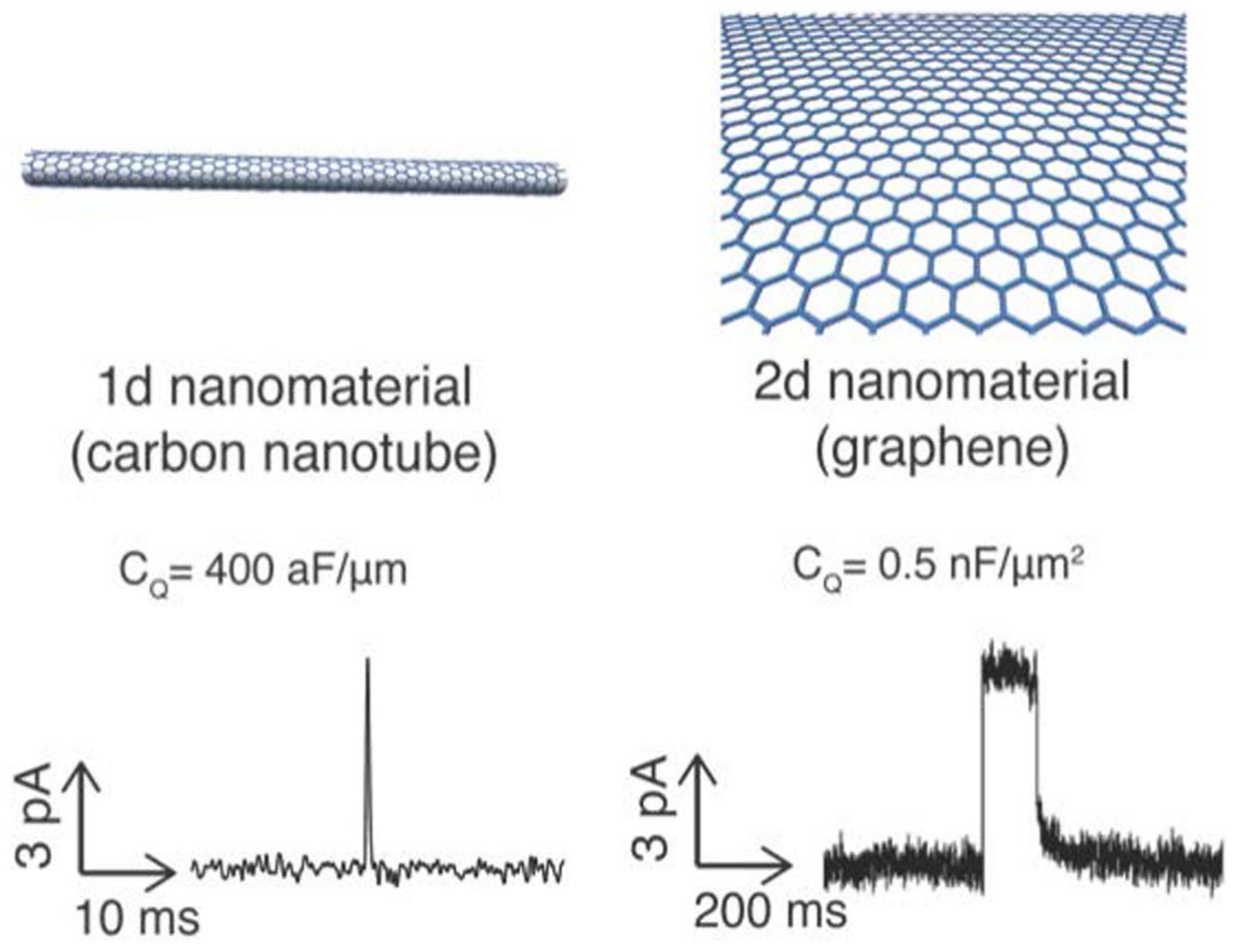
1d vs 2d ion channel measurements under nominally identical conditions (ion channel: gA; voltage: 100 mV; electrolyte 100 mM KCl). The difference in the waveforms for the two systems for identical ion channels is striking evidence of the role that dimensionality plays in the response of a nanoelectronic device to an ion channel.

If the Faradaic current is small, how, exactly, does current flow from the SWNT, through the ion channel, and into the Ag/AgCl electrode? We argue that the current is a capacitive (not Faradaic) current that is charging up the quantum capacitance of a single nanotube. A similar interpretation explained our recent analogous results with graphene^25^ instead of carbon nanotubes as the electrodes. Here, while the qualitative explanation is the same, the quantum capacitance of the nanotube is much smaller than the quantum capacitance of the graphene (e.g. ~ fF *vs* ~ μf). We argue that this is why we observe current spikes with the nanotube experiment and current steps with the graphene experiment: Under almost identical conditions, carbon nanotubes produce spikes whereas graphene produces steps in the current waveform (Fig. 6). Although these nanotube experiments use a polymer cushion that is not used by the graphene experiments, this is unlikely to be the cause of the difference in electrical properties. The addition of a polymer cushion usually can enhance the mechanical robustness and lifetime of lipid bilayers according to previous studies, but so far no study has shown that it can affect the electrical recording of ion channel activity^4^,^7^,^46^. Thus, it seems the difference in the time response is related to the dimensions and possibly the dimensionality of the material used for the electrodes.

In order to more quantitatively account for this observation, we have developed a simple physical/electrical model (based on our experience with the graphene experiments), shown in Fig. 3b, and based on the above observations as well as the observation that the size of a single gA nanopore is comparable to the size of an individual carbon nanotube. In this model, when gA opens, it locally charges up a nanotube, which has an extremely small capacitance. Since the nanotube is only weakly connected electrically to other nanotubes in the network, on the timescale of the spike (a few ms), the other nanotubes are not charged. (Note this is not a redox reaction, hence not a Faradaic current, but rather a capacitive charging which requires no physical charge transfer). In this model, once the nanotube is charged (which happens on a fast timescale compared to the few seconds that a typically ion channel stays open for), the voltage drop of the lipid bilayer is lowered to almost zero thus halting (very quickly) further current flow through the ion channel. The equivalent circuit model to explain this effect is shown schematically in Fig. 3b. Later, after a second or so has passed, the ion channel closes and the system gradually returns to equilibrium. While this model qualitatively explains the behavior of the waveform, further refinements will be needed for more quantitative relationship between the shape of the waveform and the quantum capacitance of the nanotube. For example, this model does not explain the different amplitudes, which we believe are related to the varying distance between ion channel and nanotube. In this sense, using the technique at the time it is not possible to measure accurately the *amplitude* of the current, only the *timing* (and potentially the *position*). This mechanism shows that the extremely small quantum capacitance of a 1d nanoelectronic device can be charged by a biological ion channel, an analog to our recent work showing that the quantum capacitance of 2d nanoelectronic devices also are charged as well.

What evidence is there that the activity (current spikes) detected in the gA experiments is coming from a single channel opening/closing? As in most things in science, there is no “smoking gun”, but rather an ensemble of different observations whose most likely explanation is that a single ion channel is opening and closing to cause the current spikes. First, without gA present (or alamethicin), no spikes are observed. Therefore, the current spikes are directly related to the ion channel presence in the system. Second, the amplitude of the current spikes, while not as narrowly distributed as in classical suspended lipid bilayer experiments on the same ion channel (presumably due to the varying distances between the ion channel and the particular nanotube that is addressed by it), is approximately that expected for each of the two well-studied ion channels (gA and alamethicin). Third, we never observed multiple steps, which would be indicative of more than one ion channel opening at the same time, which is statistically unlikely in the limit of few events that we have. Fourth, the functionalization scheme used does not seem to affect qualitatively the results, indicating the effect is robust. Finally, the experiments are consistent with our earlier published results using graphene electrodes and the same ion channels, where we presented additional convincing evidence that alamethicin was a single channel, by observing its 4 or 5 open configurations, which these nanotube experiments did not have enough signal to noise (in the short current spike time) to clearly observe. Taken collectively, the only reasonable explanation that is consistent with all these observations is that a single ion channel opening and closing is causing the observed current spike. That said, it is possible (and indeed likely) that each spike may come from a different ion channel, since we have so many nanotubes in our system “listening” for an event. In future experiments we will use just a single nanotube in order to further study this phenomenon.

In order to further confirm that our SLB-polymer-SWNT platform can effectively measure ion current through nanopores, we also focus on transient events in the α-HL system. α-HL is a permanent ion pore. According to our interpretation above, if the nanotube quantum capacitance is totally charged up, there should be very little voltage across the membrane, and hence very little dc current through the nanopore. The total dc current through the entire bilayer (which includes the net current through large numbers of α-HL in parallel), confirms this expectation. In addition, we did not observe any transients in the current through the α-HL pores under steady state conditions when the electrolyte solution was neat, i.e. did not contain any polymers that could block the α-HL pore.

Recently, it has been shown that polymers such as PEG^47^,^49^ and DNA^48^ can block the current through α-HL nanopores, and this has been aggressively pursued as a potential DNA sequencing technology^48^. In order to investigate the behavior of α-HL in our system, we introduced PEG into the electrolyte to observe the behavior of the current. Interestingly, we observed sharp transients (in the form of negative current spikes) in the current only when PEG was introduced into the electrolyte (Fig. 5).

In order to interpret the current transients upon introduction of PEG into the electrolyte, we consider what happens to the system when a single PEG molecule partially blocks the α-HL nanopore. In the limit of no Faradaic current, the nanotube quantum capacitance would stay charged, and the net current would not change. However, detailed circuit simulations have confirmed that, if there is a small amount of “background” Faradaic current between the nanotube and the electrolyte on the *trans* side of the chamber, (specifically if we model the CNT-electrolyte Faradaic conductance as small but not zero, i.e. 0.1 nS, consistent with literature^42^), then the nanotube quantum capacitance will discharge through this leakage current when the α-HL is blocked by the PEG. SPICE simulations confirm that, on partial blockage of the α-HL (modeling the α-HL as going from 3 nS open to 1.5 nS blocked, consistent literature values^47^,^49^), there is a negative spike of order 5–10 pA in the measured current, which is exactly what we observe^47^,^48^,^49^. Presumably, the duration of events and the amplitude of blockade correspond to the size of a polymer chain, although such a study is beyond the scope of this work and could be a completely separate, independent paper, e.g. Refs. 47, 49. In sum, this interpretation of the critical role of the nanotube quantum capacitance in the overall interaction of nanotubes with ion channels provides a single model that explains all the experimental results and demonstrates the importance of reduced dimensionality quantum effects in this new field of integrating nanoelectronics with electrophysiology at the single ion channel level.

## Discussion

In electrophysiology, measuring the ion channel currents at the cellular membrane allows one to ascertain important information about cellular activity, metabolism, cell-cell interactions, and kinetics of ion channel activities. In the current system, we have demonstrated “proof of concept” that nanotubes can be used to detect individual ion channel activity. In this system, the timing of the ion channel event is well resolved. Therefore, this SLB-polymer-SWNT platform provides a great opportunity to study cell membrane activities with high sensitivity, high temporal and spatial resolution. Furthermore, this platform combined with microfluidic channel and semiconductor-processing techniques can be integrated on wafer surface as a large-scale microarray and used for multiplexed recording. On the other hand, the spatial position still remains a challenge due to the random nature of the nanotube network.

In future work, we envision a concept of an arrayed, nanoscale resolution ion channel activity map, similar to microarray probes of ion channel activity^47^,^50^ that have recently been developed, but with the potential for true nanoscale, molecular spatial resolution. This would require additional technology development to map the network uniformity, which is currently unknown, but once such a map is developed, it could allow electrically addressable nanopore interrogation. This could find exciting applications in living systems, where a map of each ion channel activity under various physiological conditions inside a single cell or even alone a single organelle can be envisioned.

## Methods

### All-Semiconducting SWNT Deposition and Device Fabrication

Prior to nanotube deposition, the quartz wafers were treated with hot piranha solution for about an hour followed by DI water rinse to achieve a clean surface before the surface modification. The surface was treated with 1% 3-aminopropyltriethoxy silane (APTES) in isopropanol to achieve a self-assembled monolayer of an amine-terminated silane. Such amine-terminated silanes are known to enhance the selective absorption of semiconducting nanotubes. Then, purified semiconducting nanotube inks (IsoNanotubes-S 99%, Nanointegris Inc) were deposited using drop-casting method. The device array on quartz wafer was patterned by a standard photolithography process. Ti (1.2 nm)/Pd (20 nm)/Au (40 nm) were deposited by e-beam evaporation, followed by a lift-off process to form contact electrodes. The source/drain channel lengths were ranging from 2 μm to 100 μm with the width of 30 μm. To isolate metal contacts from electrolyte, lift-off photoresist (PMGI SF6, MicroChem Corp.) and Shipley S1808 (MicroChem Corp.) were coated on wafer surface as passivation layers with thickness of 300 nm and 800 nm, respectively. After another photolithography process, windows were opened only in the middle section of source/drain channel with the dimensions of 30 × 2, 30 × 15, 30 × 30 μm^2^. PDMS microfluidic channels were aligned with the device array and bonded with quartz wafer for the delivery of electrolytes with the width of 200 μm or 400 μm and the height of 50 μm.

### Polymer cushion formation

Functionalization Scheme 1: The device was incubated with 0.01 mg/ml phospholipid-PEG (DSPE-PEG) 1× phosphate buffer solution (PBS) at 4°C overnight. The hydrophobic lipid tails of the phospholipid-PEG (DSPE-PEG) are known to bond non-covalently to the hydrophobic surface of carbon nanotubes^33^, while the remaining hydrophilic tails form the hydrophilic PEG cushion to allow for lipid bilayer deposition. Thus, PEG acts as a spacer between the carbon nanotubes and the lipid bilayer.

Functionalization Scheme 2: The device was incubated with 0.01 mg/ml PLL at 4°C overnight^19^. The PLL formed a hydrophilic polymer cushion for attachment of the lipid bilayer.

Functionalization Scheme 3: Self-assembly of PEG (MW1000)-silane on SWNT device chip surface. The device was immersed into a hot DI water bath at 90°C for 1 h for increasing hydroxyl group density on quartz wafer surface before the self-assembly process. Then, the device was dried under an N_2_ stream and immersed into an ethanol solution of PEG-silane (1 mg/ml) for another 1 h to form a self-assembled layer on quartz wafer surface. After surface modification, the device was stored in a desiccator to prevent air pollution for later use. No passivation layer was applied in this functionalization process because in the self-assembly process ethanol can partially dissolve the photoresists we used for passivation layer. In order to avoid this problem, a microfluidic channel with the width of 50 μm and the height of 50 μm was placed in the center of the devices having the channel length larger than 80 μm to isolate the buffer solution from metal electrodes. To achieve a good seal, the PDMS channel was treated by O_2_ plasma (50 Watt) for 1 minute, immediately placed on PEG-silane modified chip surface by using inverted optical microscope to do alignment, and baked in oven at 60°C for 1 h.

### Lipid Bilayer formation and reconstitution of ion channels

DOPC or DPhPC (1 mM) powders were dissolved into chloroform followed by solvent evaporation overnight under a nitrogen stream to make the lipid suspension for the vesicle fusion. The dried lipid films were rehydrated in warm 1× PBS and sonicated for an hour to make small unilamellar vesicles (SUVs). Finally, the suspension was filtered by a 0.2 μm nylon filter for homogeneous SUVs which helps prevent fouling effect, and improves the quality of SLBs. The filtered lipid suspension was dropped into microchannels lying over nanotube devices and incubated for 40 min at 60°C followed by rinsing with copious 1× PBS to remove unbounded lipid bilayers. To image, DOPCs were mixed with 1 mM fluorescent dyes (Lissamine Rhodamine Red, LR-DHPE) at the molar ratio of 1000:1. To reconstitute gA into SLBs, 1 mM gA was added into DOPC chloroform solution at the molar ratio of 100:1 (final 10 μM) before the solvent evaporation process, and then mixed for two hours at room temperature followed by solvent evaporation. It is necessary to pre-mix gA with lipid molecular because there is no access to the *trans* chamber (bottom) for self-insertion of gA dimers for our device; gA dimers are normally introduced into each chamber (both the *cis* and *trans*) in traditional lipid bilayer recording systems. The voltage-gated ion channel alamethicin was incorporated into preformed lipid bilayer by directly adding to *cis* chamber (top) from its ethanolic stock resulting in a final concentration of ~5 × 10^−7^ or ~5 × 10^−6^ M. For self-insertion of α-HL, 2 μL of α-HL (0.5 mg/mL ethanolic stock) was added into cis chamber containing pure SLBs. The increase in mobility of devices started after 20 min. of addition, and the increase remained until after one hour of addition. The concentration of the gA, alamethicin, and the lipid composition were varied as described in the table in the supplementary information.

### Electrical measurement and data acquisition of ion channel recording

Ion channel recordings were performed using a patch clamp amplifier (Axopatch 200B, Axon Instruments) positioned on the vibration isolation table and shielded by a Faraday cage. All the current measurements using patch clamp system were carried out inside a Faraday cage to shield the device from external electric fields. All data were acquired and digitized by data acquisition system (Digidata 1440A, Axon Instrument). The voltage was applied to *cis* chamber through a silver/silver chloride (Ag/AgCl) wire electrode connected to the headstage of patch clamp system. The reference electrode is the SWNT network in the tran-side, which was contacted with a tungsten probe through source or drain electrode pad. Potassium chloride or cesium chloride solutions were used for measurement electrolytes (see supplementary information for detailed concentration for each experiment). A syringe pump was connected to the microfluidic channel to control the flow rate and exchange fluids. Data were low pass filtered at 1 KHz, 3 KHz using the 4-pole Bessel filter built in Axopatch 200B and sampled at 20 KHz. Data collection was done by electrophysiology software (pClamp10) and plotted using Igor Pro. The signal recording was done at voltage clamp mode with the gain of 10 mV/pA.

## Author Contributions

P.B. and T.L. conceived the original idea; W.Z., W.W. and T.L. performed the experiments and analyzed the collected data; D.J. contributed materials and T.P. contributed instrument setup; W.Z., W.W., T.L. and P.B. wrote the manuscript; All authors reviewed the manuscript.

## Supplementary Material

Supplementary Information Detection of single ion channel activity with carbon nanotubes
