# Supplementary material for: Detection of single ion channel activity with carbon nanotubes
Source: Sci Rep. 2015 Mar 17;5:9208. doi: 10.1038/srep09208 (PMC4361846; doi:10.1038/srep09208)
Supplement: Supplementary Information — Detection of single ion channel activity with carbon nanotubes [file srep09208-s1.doc]

**Supplementary Information**

**Title: Detection of single ion channel activity with carbon nanotubes**

**Author**: Weiwei Zhou, Yung Yu Wang, Tae-Sun Lim, Ted Pham, Dheeraj Jain, Peter J. Burke

***Author affiliation***: Integrated Nanosystems Research Facility, Department of Electrical Engineering and Computer Science, University of California Irvine, Irvine, CA, 92697 USA

***Corresponding author***: *Peter J. Burke*, pburke@uci.edu, Phone: (949) 824-9326, Fax: (949) 824-3732


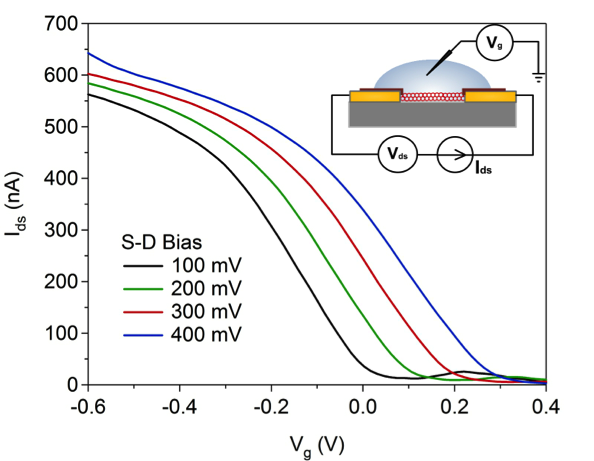


**Figure S1**. **Depletion curves of bare nanotube devices.** Typical p-type depletion curves of the bare nanotube transistor in 150 mM KCl solution with different source-drain (S-D) biases, indicating that the device is working properly in aqueous solution with on/off ratio of ~1000 and transconductance of ~1.2 μS. Positive threshold voltage shifts were observed due to increasing positive S-D bias, inset shows a device set-up.

**Figure S2**. **Depletion curves of a control sample device.** There is no carbon nanotube between source and drain electrodes.

**
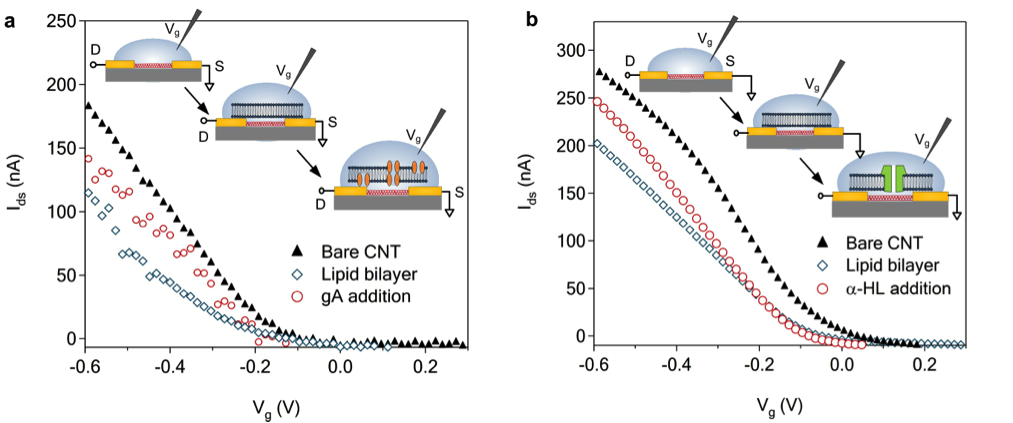
**

**Figure S3** **Effect of lipid bilayers and ion channels on device characteristics.** (a) Depletion curves recorded with bare nanotube transistor (black), with lipid bilayers (blue) and after gA addition (red), V_ds_=10 mV. (b) Depletion curves with lipid bilayer before (blue) and after introduction of α-HL (red) showing an increase in mobility; V_ds_=10 mV. Inset: Graphics of device configurations.


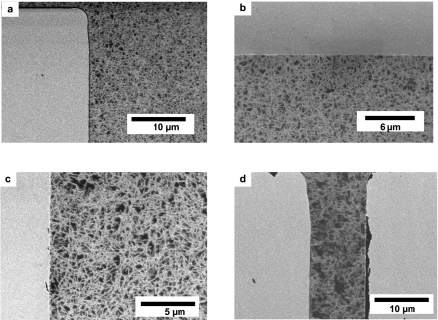


**Figure S4. Uniform nanotube networks.** SEM images of nanotube networks in four different locations of gate channel area at (a), (b), (c), (d). The density of CNT networks created can be controlled by adjusting the concentration of the nanotube ink. In this study, the nanotube density for all devices kept constant at 5~10 nanotube µm^-2^ and uniform as shown in SEM images.


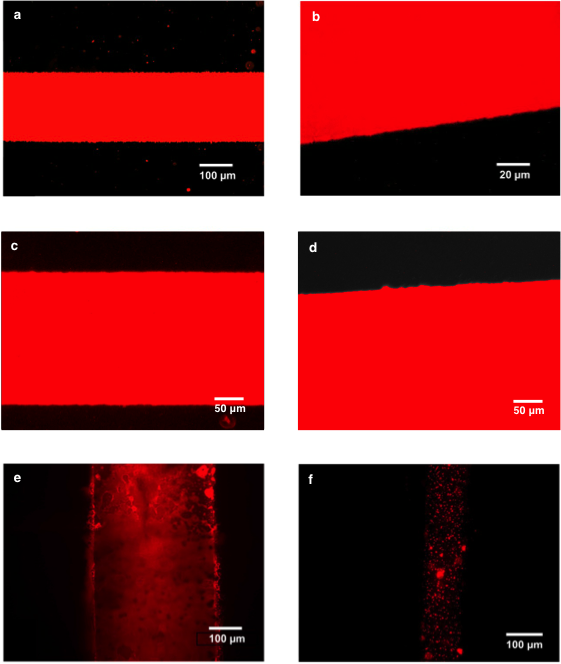


**Figure S5. Lipid bilayer depositions**. Fluorescence images of lipid bilayers (DOPC) on various substrates conditions where SLBs are confined by microfluidic channels (a) lipid bilayer (red) on a glass substrate carefully treated with hot piranha, (b, c and d) lipid bilayers (red) incorporated with gA on PEG-lipid, PLL and PEG-silane respectively functionalized CNT networks, (e) lipid bilayers (red) on non-functionalized CNT networks displaying not perfectly continuous SLB films with several dark areas which indicate no or less SLBs (f) lipid bilayer (red dots) incorporated with gA on non-functionalized CNTs showing non-uniform and poor coverage.

**Yield/statistics**

Overall the experiments had a yield (i.e. observation of spikes in the current due to single ion channel events) of about 60% (10 out of 16 independent experiments, see table below). Overall when current spikes occurred, their amplitude was about what was expected for that ion channel (gA or alamethicin).

| Ion channel | Concentration (gA or Al) | Electrolyte | Voltage | Functionaliz-ation Scheme | Lipid composition | Current Spikes | Spike amplitude |
| --- | --- | --- | --- | --- | --- | --- | --- |
| gA | 0.02 mg/ml | 100 mM KCl | 50 mV | Lipid-PEG | DOPC | Yes | 10pA |
| - (Control) | 0 | 100 mM KCl | 50 mV | Lipid-PEG | DOPC | No | - |
| gA | 0.02 mg/ml | 100 mM KCl | 100 mV | Lipid-PEG | DOPC | No | - |
| gA | 0.02 mg/ml | 1 M KCl | 10 mV | Lipid-PEG | DOPC | No | - |
| gA | 0.04 mg/ml | 1 M CsCl | 200 mV | Poly-L-lysine | DOPC | Yes | 6 pA |
| gA | 0.2 mg/ml | 1 M CsCl | 200 mV | Poly-L-lysine | DOPC | No | - |
| gA | 0.02 mg/ml | 1 M CsCl | 200 mV | Poly-L-lysine | DOPC | Yes | 10 pA |
| gA | 0.02 mg/ml | 1M CsCl | 200 mV | Poly-L-lysine | DOPC | Yes | 20 pA |
| gA | 0.02 mg/ml | 100 mM KCl | 100 mV | Lipid-PEG | DOPC | Yes | 3-4 pA |
| gA | 0.02 mg/ml | 100 mM KCl | 100 mV | Lipid-PEG | DPhPC | No | - |
| - (Control) | 0 | 100 mM KCl | 100 mV | Lipid-PEG | DPhPC | No | - |
| gA | 0.02 mg/ml | 100 mM KCl | 100 mV | PEG-silane | DPhPC | Yes | 10-15 pA |
| gA | 0.02 mg/ml | 100 mM KCl | 100 mV | PEG-silane | DPhPC | Yes | 10-15 pA |
|  |  |  |  |  |  |  |  |
| Al | 1 μg/ml | 100 mM KCl | 180 mV | Lipid-PEG | DOPC | Yes | 100 pA |
| Al | 10 μg/ml | 100 mM KCl | 180 mV | Lipid-PEG | DOPC | No | - |
| Al | 10 μg/ml | 100 mM KCl | 180 mV | Lipid-PEG | DOPC | No | - |
| Al | 10 μg/ml | 100 mM KCl | 100 mV | PEG-silane | DPhPC | Yes | 100 pA |
| Al | 10 μg/ml | 100 mM KCl | 100 mV | PEG-silane | DPhPC | Yes | 100 pA |

.
